# Genome-Wide Survey and Comparative Analysis of Long Terminal Repeat (LTR) Retrotransposon Families in Four *Gossypium* Species

**DOI:** 10.1038/s41598-018-27589-6

**Published:** 2018-06-20

**Authors:** Zhen Liu, Yuling Liu, Fang Liu, Shulin Zhang, Xingxing Wang, Quanwei Lu, Kunbo Wang, Baohong Zhang, Renhai Peng

**Affiliations:** 1 Anyang Institute of Technology, Anyang, Henan 455000 China; 2 State Key Laboratory of Cotton Biology/Institute of Cotton Research of Chinese Academy of Agricultural Science, Anyang, Henan 455000 China; 3 Department of Biology, East Carolina University, Greenville, NC 27858 USA

## Abstract

Long terminal repeat (LTR) retrotransposon is the most abundant DNA component and is largely responsible for plant genome size variation. Although it has been studied in plant species, very limited data is available for cotton, the most important fiber and texture crop. In this study, we performed a comprehensive analysis of LTR retrotransposon families across four cotton species. In tetraploid *Gossypium* species, LTR retrotransposon families from the progenitor D genome had more copies in D-subgenome, and families from the progenitor A genome had more copies in A-subgenome. Some LTR retrotransposon families that insert after polyploid formation may still distribute the majority of its copies in one of the subgenomes. The data also shows that families of 10~200 copies are abundant and they have a great influence on the *Gossypium* genome size; on the contrary, a small number of high copy LTR retrotransposon families have less contribution to the genome size. Kimura distance distribution indicates that high copy number family is not a recent outbreak, and there is no obvious relationship between family copy number and the period of evolution. Further analysis reveals that each LTR retrotransposon family may have their own distribution characteristics in cotton.

## Introduction

Transposons are mobile genetic elements that can be multiplied in the genome using a variety of mechanisms. Transposons play crucial roles in gene expansion, diversification and evolution^1,2^. Additionally, they are major determinants of genome size in eukaryotes. Indeed, there is a linear correlation between genome size and transposable element content in all eukaryotes^3–5^. The most numerous group of transposons in plants are long terminal repeat (LTR) retrotransposon, which share a unique structural feature, two long terminal repeats, and they typically contain ORFs for a structural protein for virus-like particles, aspartic proteinase, reverse transcriptase, RNase H, and integrase. Occasionally, there is an additional ORF of unknown function^6^. Two main super families of LTR retrotransposon are Gypsy and Copia, differing in the order of reverse transcriptase and integrase^2^. An LTR retrotransposon family is a group of elements that have high DNA sequence similarity in their coding region or internal domain, or in their terminal repeat region. According to Wicker^2^, the sequence similarity was higher than 80%. The lowest taxon of LTR retrotransposon is a particular individual copy, corresponding to a specific transposition and insertion event, and is of particular relevance to genome annotation^2^.

Cotton (*Gossypium* spp.) is a remarkably diverse genus, with over 50 recognized species divided into 8 diploid genome groups and a single, monophyletic allopolyploid lineage^7^. It has been proposed that all diploid cotton species might evolve from a common ancestor that subsequently diversified to 8 groups, including groups A–G and K^8,9^. DNA sequence data place the origin of *Gossypium* at about 5 to 10 million years ago, which rapidly diversified into these major genome groups shortly thereafter. Allopolyploid species appeared within the last 1 to 2 million years, which were involved a D-genome species as the pollen-providing parent and an A-genome species as the maternal parent^8,9^. *Gossypium* has undergone a threefold increase in genome size due to the accumulation of LTR retrotransposons since its origin^10^. Further studies show that retrotransposable-mediated gene regulation may modulate the unbalanced expression of homologous gene pairs that results in that great differences in fiber properties among *Gossypium* species^11,12^.

Recent whole genome sequencing of two cultivated tetraploid species, island cotton (*Gossypium barbadense*)^13^ and upland cotton (*G. hirsutum*)^14^, and their two diploid ancestors *G. arboretum*^15^, and *G. raimondii*^16,17^ make it possible for detailed discovery and cross-species comparison of LTR retrotransposons elements at the whole genome level^13,15–20^. In this study, we annotated the LTR retrotransposon of four *Gossypium* species (*G. barbadense*, *G. hirsutum*, *G. arboreum* and *G. raimondii*) by using multiple de novo repeat prediction pipelines with a combination of known repeat elements from the RepBase database^21^. For the first time, We classified the LTR retrotransposon sequences into the family level, and we also compared the LTR retrotransposon families between *G. barbadense*^13^, *G. hirsutum*^14^, *G. arboreum*^15^ and *G. raimondii*^16^. Our results led to the identification of several previously unknown features of *Gossypium* LTR retrotransposon families associating with copy number, genomic distribution, average element size, and the correlation with the genes. Our results highlight the importance of distinguishing LTR retrotransposon families instead of super families when assessing their impact on gene and genome evolution.

## Methods and Materials

### Dataset

Genome sequences and annotations of two cultivated tetraploid species, island cotton (*G. barbadense*)^13^ and upland cotton (*G. hirsutum*)^14^, and their two diploid ancestors *G. arboretum*^15^, and *G. raimondii*^16,17^ were downloaded from the cotton public available database COTTONGEN^22^. The genomes of *Theobroma cacao*, *Carica papaya*, *Arabidopsis thaliana*, *Populus trichocarpa*, *Ricinus communis*, *Durio zibethinus* and *Glycine max* were download from NCBI (ftp.ncbi.nlm.nih.gov/).

### LTR retrotransposon identification

Self-comparison approaches were performed by PILER^23^. Because the genome is too large to align the entire sequence to itself, the genome was split into chunks small enough for PALS^23^. Each chunk was aligned to itself, then each different pair of chunks was aligned to each other. Signature-based identification of LTR retrotransposon elements were performed using LTR_STRUC^24^ and LTRharvest^25^ with the default parameters. Then, all putative LTR retrotransposon elements were classified into super families (such as Copia and Gypsy) by REPCLASS^26^. Within each super family, CD-HIT^27^ was used to cluster similar sequences into groups if they shared at least 80% of sequence identity and consensus sequences of every group were created. In addition, the de-novo repeat identification suite RepeatModeler^28^ was also used to produce the consensus sequences. A library was established by integrating these consensus sequences with Repbase^21^ and the redundant sequences were removed from the library if they shared 80% sequence identity with any sequence in Repbase. Then, genomes of *G. barbadense*, *G. hirsutum*, *G. arboretum*, and *G. raimondii* were annotated by RepeatMasker^29^ with this custom library. In order to analyze the origin of LTR retrotransposon, genomes of *T. cacao*, *C. papaya*, *A. thaliana*, *P. trichocarpa*, *R. communis*, *D. zibethinus* and *G. max* were also annotated by RepeatMasker with the same library. A set of sequences annotated by the same consensus sequence of the library were defined as a LTR retrotransposon family. A family should consist of at least 10 elements. In this way, more families will be retained so as to understand the characteristics of the low copy family.

### Structural analysis

LTR_STRUC was employed to generate a variety of structural features of LTR retrotransposon. By using blastn^30^, family elements that were generated by other methods were aligned against LTR_STRUC results. For analyzing the structures of LTR retrotransposon, a set of perl script was developed.

### Copy number and genome coverage estimation

Copy number and genome coverage of LTR retrotransposon were calculated on RepeatMasker outfiles (.out) using custom perl scripts. The calculation only included sequences longer than 100 nucleotides and short sequences were eliminated. Additionally, if multiply significant hits were detected for a same genome location, only the longer sequence was considered. The distributions and densities of genes were obtained from the GFF3 annotation.

*Gossypium* genomes were split into contiguous 2-Mb windows. An LTR retrotransposon sequence or gene was assigned to a particular window based on its start point. Circos^30^ was employed for constructing the diagram. To understand the function of the genes around LTR retrotransposon family, we collected genes locating within both upstream and downstream 20 kb of the LTR retrotransposon family according to the position of transposon and GFF3 annotation files. GO annotation results were plotted by WEGO^31^.

### Kimura distance distribution of LTR retrotransposon elements

Kimura distances^32^ between LTR retrotransposon elements and its consensus sequence from the library were determined using the RepeatMasker alignments result file (.align).

### Phylogenetic analysis

Sequence alignments were performed by Clustal Omega program^33^ with default options. PHYLIP 3.695 program^34^ was employed for the neighbor-joining trees.

## Results

### LTR retrotransposon families in cotton genome

Using a combination of the previously-known sequences from RepBase and newly identified by multiple de novo methods, a *Gossypium* species-specific LTR retrotransposon library was constructed. The library was composed of 78468 consensus sequences, 38839 of which were classified as Gypsy super family, 11031 were classified as Copia super family, 7250 were classified as other super family, and 21348 could not be classified. Of these consensus sequences, 54649 were identified de novo and 23819 were from Repbase.

**Figure 1:**
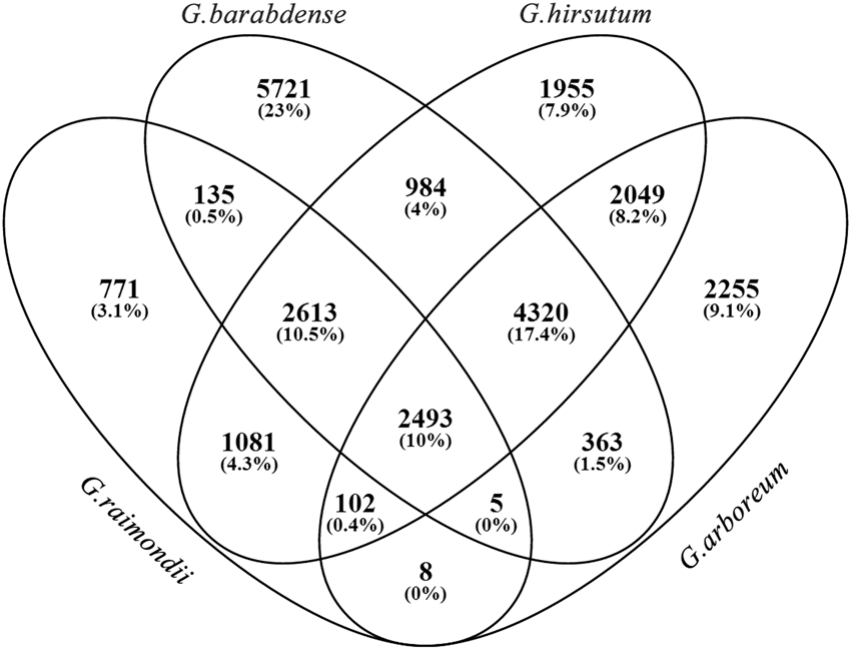
Comparison of LTR retrotransposon families between *Gossypium* genome.

**Figure 2:**
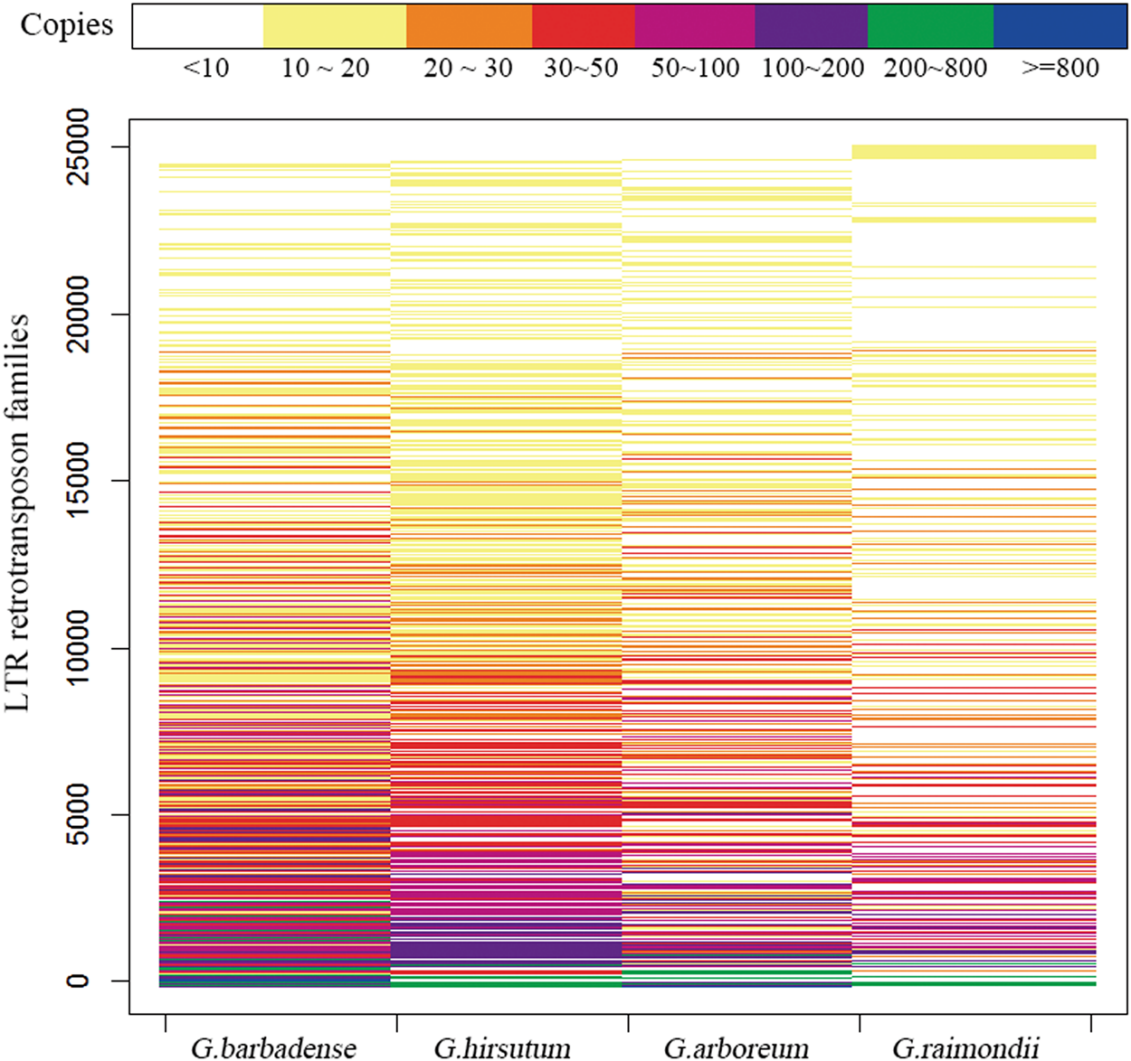
Copy number differences of the same family in different species.

We statistically analyzed the family number of LTR retrotransposons in *Gossypium* genomes on condition that the family must contain at least 10 copies and the elements must be longer than 100 bp. After such filtering, a total of 24855 families have been identified; *G. barabdense*, *G. hirsutum*, *G. arboreum*, and *G.raimondii* contain 16634, 15597, 11595, 7208 families, respectively (Supplementary Table S1). The number of LTR retrotransposon families shared between the *Gossypium* species was shown in Venn^35^ diagram (Fig. 1). Among them, 2493 (10%) families were shared by all the four *Gossypium* species, and 10702 (43.1%) families were specific to one specific species; for example, *G. barabdense* contains 5721 (23%) specific families. In general, most high copy number LTR retrotransposon families are highly copied in all of the four *Gossypium* species, and most low copy number families have few elements present in all of four *Gossypium* species (Fig. 2).

LTR retrotransposon elements were highly unstable in plant genomes, and the instability was associated with illegitimate recombination and unequal homologous recombination. In our study, there are 102 families that are present in *G. hirsutum*, *G. arboreum*, and *G.raimondii* but not in *G. barbadense*. Structural analysis of these 102 families shows that 79.0%, 75.4% and 82.3% of these elements in *G. hirsutum*, *G. arboreum*, and *G. raimondii* are remnants fragments that were derived from illegitimate recombination. Similar results were obtained from families that only absent in *G. hirsutum*, *G. arboreum* and *G. raimondii* (Fig. 1), that about three-quarter of these elements are remnants fragments. We also found families specific to one of the four *Gossypium* species (Fig. 1). These families might be originated from duplication events after the *Gossypium* species separated or might be caused by the loss of the other three species during *Gossypium* evolution. Comparative analysis shows that only a small part of these families had members in *T. cacao*, *C. papaya*, *A. thaliana*, *P. trichocarpa*, *R. communis*, *D. zibethinus* and *G. max* genomes, that is to say most families originate after the separated of *Gossypium* species.

### Evolution of LTR retrotransposon family during cotton polyploidization

**Figure 3:**
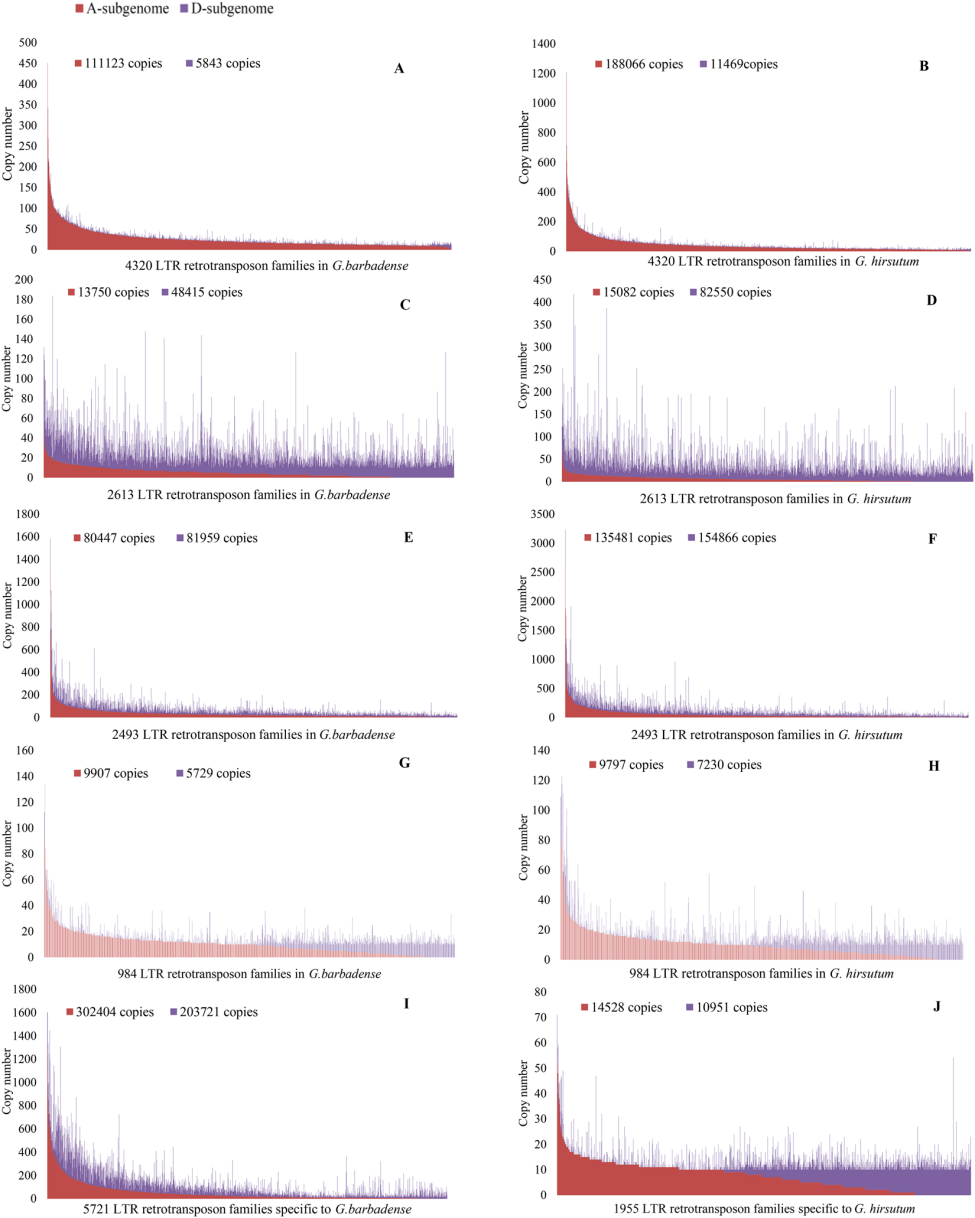
Copy number distribution in A- and D-subgenomes of the tetraploid genomes.

Polyploidy is a pervasive force in plant evolution. The AD-genome tetraploid *G. barbadense* and *G. hirsutum* were derived from progenitor A-genome and D-genome diploids involved in ancestral allopolyploidization. In this study, to understand the evolution of tetraploid *Gossypium*, we calculated the distribution preferences of LTR retrotransposon families that origin in different evolution stage. There are 4320 LTR retrotransposon families shared by tetraploid *G.barbadense, G. hirsutum* and A-genome diploid *G. arboreum* (Fig. 1). However, none of these families originated from D-genome. For these families, there are 111123 copies in A-subgenome and only 5843 copies in D-subgenome in *G. barbadense* (Fig. 3A). In addition, there are 2613 LTR retrotransposon families shared by *G. barbadense, G. hirsutum* and D-genome diploid *G. raimondii* (Fig. 1). The LTR retrotransposon family copies also show unequal distribution in A- and D-subgenome (Fig. 3C,D). In order to make a comparative analysis, we calculated the distribution of 2493 LTR retrotransposon families that shared by the two tetraploid genomes and their A- and D-genome diploid ancestors. The results show that these transposons were approximately equal distributed in the A- and D-subgenomes of the tetraploid genomes (Fig. 3E,F). Therefore, in tetraploid *Gossypium* species, LTR retrotransposon families from the progenitor D genome may have more copies in D-subgenome, and families from the progenitor A genome may have more copies in A-subgenome.

We also calculated the distribution of tetraploid specific LTR retrotransposon families. First of all, there are 984 families shared by *G. barbadense* and *G. hirsutum* (Fig. 1). Secondly there are 1955 families specific to *G. hirsutum* (Fig. 1). Interestingly, the majority of these transposon families had a tendency to be distributed either in A-subgenome (Left of the figure) or in D-subgenome (Right of the figure, Fig. 3G,H and J). Third, there are 5721 families specific to *G. barbadense* (Fig. 1), whereas no great preference was detected and there are 302404 copies in A-subgenome and 203721 copies in D-subgenome (Fig. 3I). Therefore, LTR retrotransposon families that insert after polyploid formation may still distribute most of its copies in one of the subgenomes or both subgenomes.

### Copy number characterization of LTR retrotransposon family

**Figure 4:**
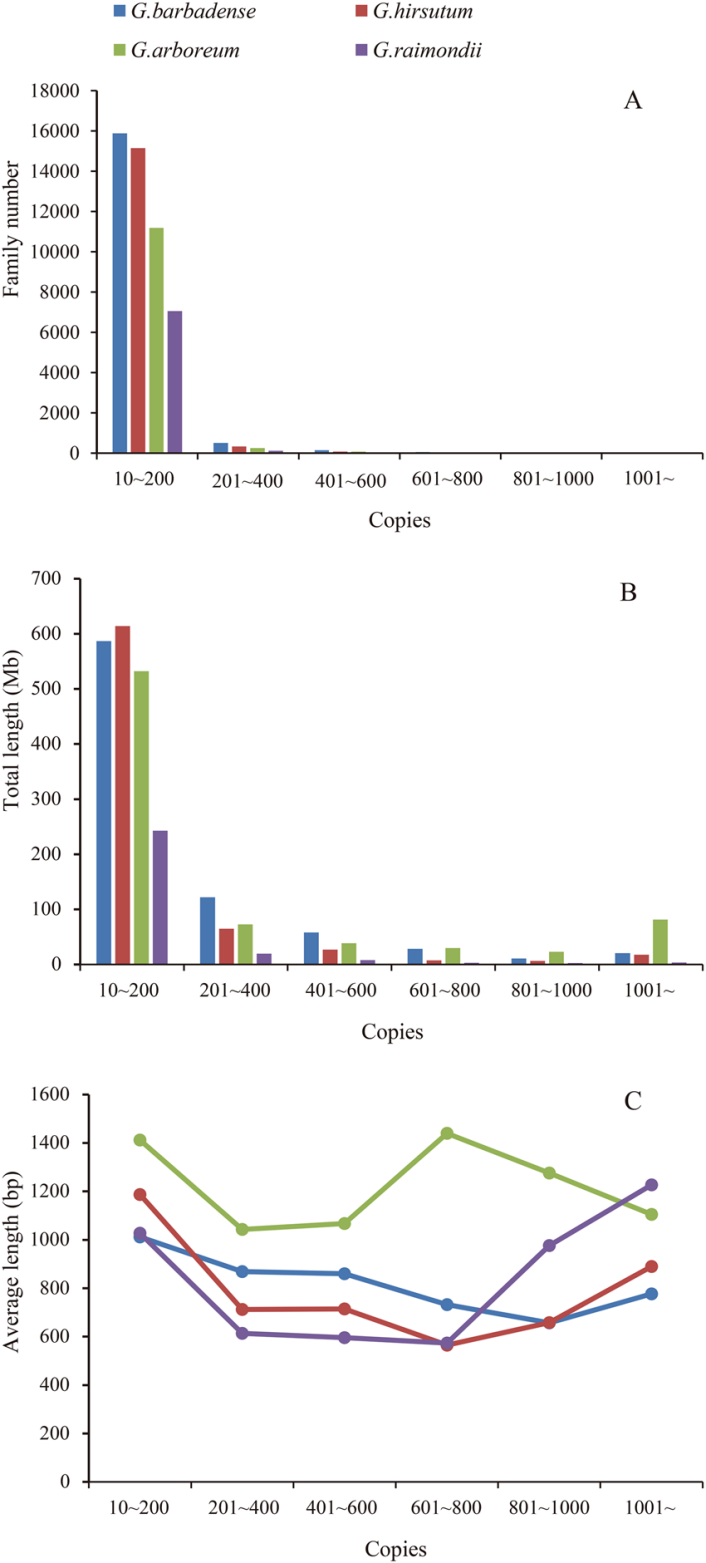
Family number, total length and average length of different copy number LTR retrotransposon families.

LTR retrotransposon families with 10–200 copies are most abundant, and families of more than 800 copies are very rare (Fig. 4A). For example, in the *G. hirsutum* genome, 15143 LTR retrotransposon families have the copy number between 10 and 200, but only 23 LTR retrotransposon families show the copy number with 800 or more. Interestingly, there is a family whose copy number is significantly higher than others in *G. hirsutum*, *G. arboreum* and *G. raimondii* genomes. In both *G. hirsutum* and *G. arboreum* genome, the highest copy numbers family RLGy_42738 has 3229, 10055 copies, respectively, followed by the family of 1913, 3830 copies. In *G. raimondii* genome, the highest copy numbers family RLGy_31317 has 1995 copies, followed by family of the 1076 copies. However, there is not so much difference between the high copy number families in the *G. barbadense* genome (Supplementary Datasets 1–5). Furthermore, there are five LTR retrotransposon families whose copy number is more than 400 in all of the four *Gossypium* genomes (Table 1).

**Table 1:** Copy number of the five LTR retrotransposon families that shared by all of the four *Gossypium* genomes.

	*G. raimondii*	*G. barbadense*	*G. hirsutum*	*G. arboreum*
RLGy_42738	515	1587	3229	10055
RLCo_3154	857	788	1743	1691
RLCo_5655	625	538	1619	1952
RLGy_42774	459	939	1363	1122
RLCo_258	711	468	1188	907

Large amount of LTR retrotransposon families with 10~200 copies in the *Gossypium* genome resulting in the total length of these families are the most abundant. In the *G. hirsutum* genome, LTR retrotransposon families of this copy level is 614.1 Mb, whereas families with 800 or more copies is only 24.1 Mb. These findings are similar in the other three *Gossypium* genomes (Fig. 4B). Therefore, LTR retrotransposon families with 10~200 copies have a great influence on the genome size, while a small number of high copy LTR retrotransposon families have less contribution to the *Gossypium* genome size.

The average length of the LTR retrotransposon family can reflect the degree of fragmentation, which may reflect some evolutionary characteristics. In *G. arboreum* genome, average length of LTR retrotransposon families with 600~1000 copies show little longer. In these families, RLGy_40347 family has 659 copies, with an average length of 9271 bp; RLGy_40817 family has 908 copies, with an average length of 8123 bp. In addition, the average length of RLGy_40479 is 9496 bp, which is the longest among all studied families, with a copy number of 1040. The above three families are all *G. arboreum* specific. Altogether, there is not much difference in the average length of different copy number families (Fig. 4C).

### Distribution of LTR retroelements on chromosomal

**Figure 5:**
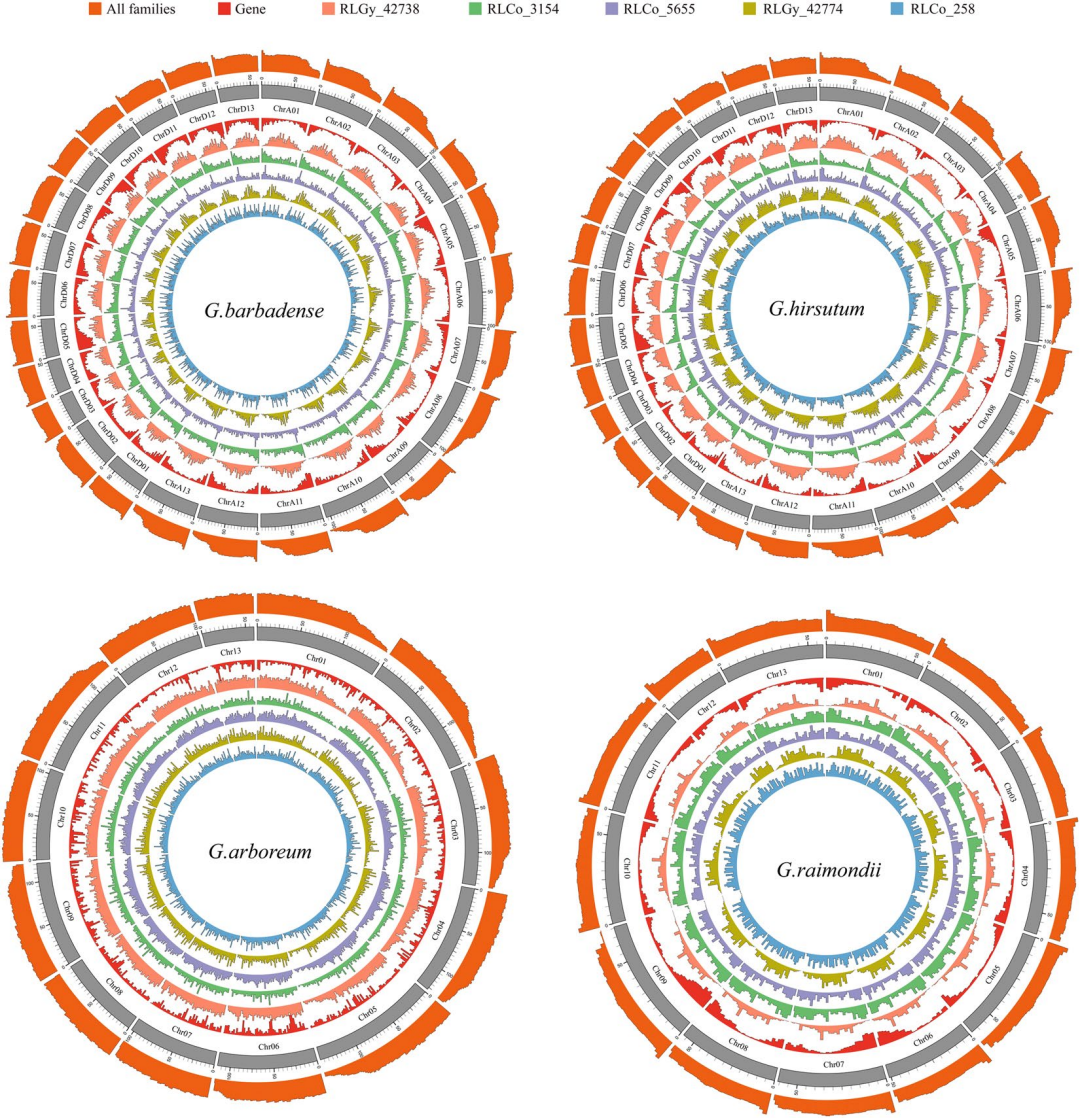
LTR retrotransposon family elements and gene density of *Gossypium* chromosomes in 2 Mb unit. The outermost ring is the density of all LTR retrotransposon families elements, after the chromosome ring, followed by RLGy_42738 family, RLCo_3154 family, RLCo_5655 family, RLGy_42774 family and RLCo_258 family.

LTR retroelement elements are ubiquitous in *Gossypium* genomes. To be more precise, they are less densely distributed at the end of the A-genome chromosomes, more densely at the head of the D-genome chromosomes. In addition, uniform distribution on the chromosomes 7, 8 10, 12 and 13 of *G. arboretum* and chromosome A4 of *G. hirsutum* (Fig. 5).

In order to understand the distribution pattern of each LTR retroelement family in *Gossypium* genomes, we analyzed 5 families that were shared by all of the four *Gossypium* species and the copy number is more than 400 (Table 1). In *G. barbadense*, *G. hirsutum* and *G. raimondii*, RLGy_42738 and RLGy_42774 show approximate negative correlation with gene density; however, RLCo_3154 and RLCo_5655 show approximate positive correlations with gene number at the head of the chromosomes. Whereas the distribution of these families in *G. arboreum* genome show inconsistent correlations with the other three *Gossypium* genomes. In addition, the distribution of RLCo_258 in all of the four *Gossypium* genomes had no obvious relationship with gene density, but it had its own unique characteristics and was obviously not random (Fig. 5).

The GO clustering analysis show that the same family in different species and different families in the same species differed. However, in total, the majority of the genes around RLGy_42738, RLCo_3154, RLCo_5655, RLGy_42774 and RLCo_258 under cellular component were involved in cell and cell part, molecular function categories were mostly clustered in binding and catalytic, the genes clustered under biological process mainly involved in cellular process and metabolic process (Supplementary Fig. S1).

### Copy number of LTR retroelement family and evolution

**Figure 6:**
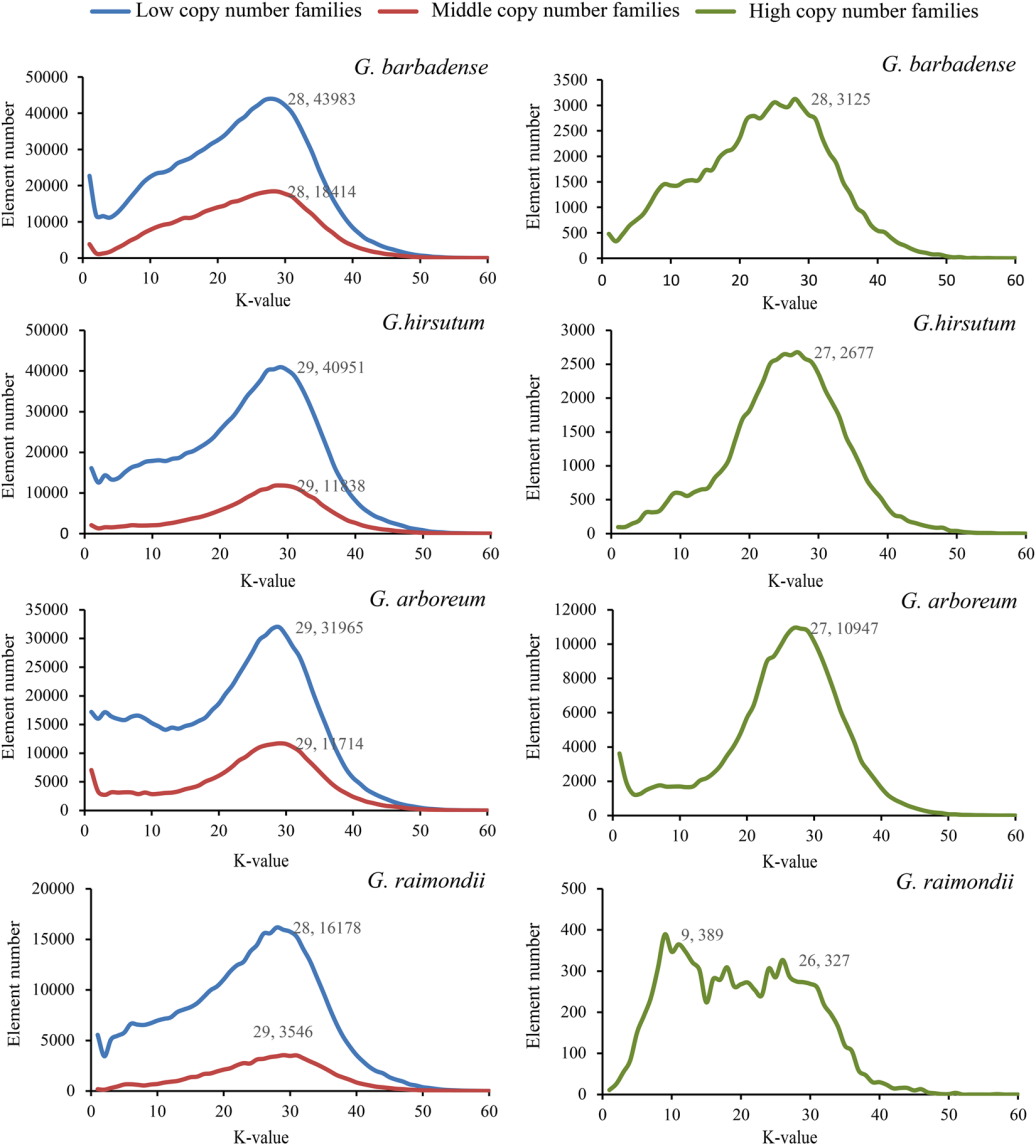
Kimura distance distribution of LTR retrotransposon. The graphs represent element number (y axis) for high copy number families (>800 copies), middle copy number families (201~800) and low copy number families (10~200) in *Gossypium* genomes (x axis, K-value from 0 to 60). Due to the large difference, high copy number families was separated from middle copy number families and low copy number families.

Kimura distances were calculated for all copies of LTR retroelement family by RepeatMasker. Sequence divergence (K-values) is correlated with the transposition history: low K-values copies indicate recent activity, while high K-values elements were generated by more ancient transposition events^32^. In this study, we generally separated LTR retroelement families into high copy number families (>800 copies), middle copy number families (201~800 copies) and low copy number families (10~200 copies). Profiles were relatively similar not only between species but also between different copy number groups (Fig. 6). First, Despite the dramatic copy number differences across species, all of the four *Gossypium* genomes had the most LTR retroelement copies at about k = 28; Second, *Gossypium* genomes generally contain much more recent copies (k < 28) than ancient copies (k > 28). We speculate that LTR retrotransposon elements were eliminated from the genome before the evolution stage of k > 28. This is in agreement with the dynamics of LTR retrotransposons in the rice genome^36^. Furthermore, a young (k < 3) and small amplification of LTR retrotransposon elements was occurred in *Gossypium* genomes, such as the low copy number families of *G. barbadense*, middle and high copy number families of *G. arboreum*. In addition, *G. raimondii* high copy number families have a regular amplification over time (k < 26). These young bursts vary among species, revealing that each genome has undergone a particular amplification within very recent evolution history, which may be related to their growth environment.

The results also show that the high copy number families were not a recent outbreak, but like other families that there are more elements distribute near k = 28. Therefore, there was no significant relationship between the copy number and the evolutionary period.

### Phylogenetic analysis

The phylogenetic relationships of the five shared LTR retroelement families (Table 1) among the four *Gossypium* species further reflected by the tree individually. The results show that all clades are mixed of elements from the four *Gossypium* genomes, certain elements are more closely related to copies present in other *Gossypium* species than to the sequences of self-genome (Supplementary Fig. S2).

## Discussion

### Comparative analysis of tetraploid Gossypium and its diploid progenitors

Allopolyploidization plays a profound impact on genome architecture^37^. Some of these changes have been linked to novel phenotypic variations in polyploids^38^. Relative to diploid progenitor cottons, tetraploid cottons (*G. hirsutum* and *G. barbadense*) developed superior fiber phenotypes, representing the development of new agronomic traits^13^. In *G. hirsutum* genome^14^, overall gene order and colinearity are largely conserved between the A and D subgenomes and the extant D-progenitor genome (*G. raimondii*). However, this colinearity was not obvious with the A-progenitor genome^14^. Furthermore, SNP analysis indicated that the genomic divergence between the two tetraploids was less than the divergence between the tetraploid and its diploid progenitors^13^. Our analysis shows that the shared families (10410) between tetraploids (*G. barbadense* and *G.hirsutum*) were more than the shared families between the tetraploid and its diploid progenitors (Fig. 1). *G. hirsutum* genome shares 6289 families with *G. raimondii* and shares 8964 families with *G. arboretum*; *G.barbadense* genome shares 5264 families with *G. raimondii* and shares 7181 families with *G. arboretum*. This study is an important step in the analysis of the tetraploid cotton genome, which will provide invaluable insights into the biology and evolution of allopolyploidization in cotton and other plants.

### Conserved features of LTR retrotransposon

Copy numbers of shared LTR retrotransposon families differ among the four *Gossypium* genomes (Supplementary Table S1). In addition, the total amount of LTR retrotransposon elements is also quite different. However, copy number and average length characteristics of the LTR retrotransposon family are similar in different *Gossypium* species (Figs 2 and 4). Furthermore, many researches show that larger genomes tend to have more LTR retrotransposons than smaller genomes^3^, and we also observed a similar result in *Gossypium* genomes, except for *G. arboreum* (Table 2). These features of LTR retrotransposon indicate their importance to the genome evolution.

**Table 2:** Transposon length and genome size of the four *Gossypium* species.

	*G. raimondii*	*G. arboreum*	*G. hirsutum*	*G. barbadense*
LTR retrotransposon	279.1 Mb	777.8 Mb	737.3 Mb	827.2 Mb
Genome	752.8 Mb	1694 Mb	2491.1 Mb	2513.1 Mb
% of genome	37.07	45.91	29.60	32.92

### Distribution characteristics

A large number of transposable elements have been identified from genomes of different species. However, the transposable element distribution shows great or even the opposite characteristics. In maize and sorghum, transposable elements had a strong positive correlation with gene number and have a bias toward insertion near genes, but with a preference for avoiding coding regions^39^. On the contrary, in *Arabidopsis*, transposable element distribution analysis suggests a negative correlation between gene and TE density^40^.

In this study, we annotated the LTR retrotransposon of four *Gossypium* species and classified these elements into the family level for the first time. One of the most interesting findings is the observation that the distribution of LTR retrotransposon elements had no insertion bias as a whole, however, each family had its own unique distribution characteristics (Fig. 5). Furthermore, if we exclude the special *G. arboreum*, the same family has similar distribution characteristics in *G. barbadense*, *G. hirsutum* and *G.raimondii*. If so, the distribution characteristic is another conserved feature of LTR retrotransposon families.

## Conclusions

The current understanding of LTR retrotransposon increases with an increasing number of sequenced genomes from a broad taxa range. In addition to Arabidopsis^41^, great progress has been made in LTR retrotransposon studies in corn^37^, soybean^42^ and tomato^43^. In the present study, we identified and classified LTR retrotransposon from genomes of *G. barbadense*, *G. hirsutum*, *G. arboreum* and *G. raimondii*. Our results suggest that most LTR retrotransposon families have 10~200 copies in the genome. Additionally, there is no obvious relationship between family copy number and the period of evolution. By analyzing 5 of the 2493 shared families, we found that different families have different distribution characteristics; more importantly, the same family has similar distribution character across *G. barbadense*, *G. hirsutum* and *G. raimondii*^44,45^.

## Electronic supplementary material

Supplementary Figures

Supplementary Datasets
