# Supplementary material for: Genome-Wide Survey and Comparative Analysis of Long Terminal Repeat (LTR) Retrotransposon Families in Four Gossypium Species
Source: Sci Rep. 2018 Jun 20;8:9399. doi: 10.1038/s41598-018-27589-6 (PMC6010443; doi:10.1038/s41598-018-27589-6)
Supplement: Supplementary file 1 — Supplementary Figures [file 41598_2018_27589_MOESM1_ESM.pdf]

Supplementary Figures to:

Genome-Wide Survey and Comparative Analysis of Long Terminal Repeat (LTR) Retrotransposon Families in Four *Gossypium* Species

Zhen Liu [1], Yuling Liu [1], Fang Liu [2], Shulin Zhang [1], Xingxing Wang [2], Quanwei Lu [1], Kunbo Wang [2], Baohong Zhang [3,*] and Renhai Peng [1,2,*]

[1]Anyang Institute of Technology, Anyang, Henan, 455000, China

[2]State Key Laboratory of Cotton Biology/Institute of Cotton Research of Chinese Academy of Agricultural Science, Anyang, Henan, 455000, China

[3]Department of Biology, East Carolina University, Greenville, NC, 27858, USA

Running title: LTR retrotransposon families in cotton

[*]Corresponding authors: Baohong Zhang (zhangb@ecu.edu); Renhai Peng (aydxprh@163.com)

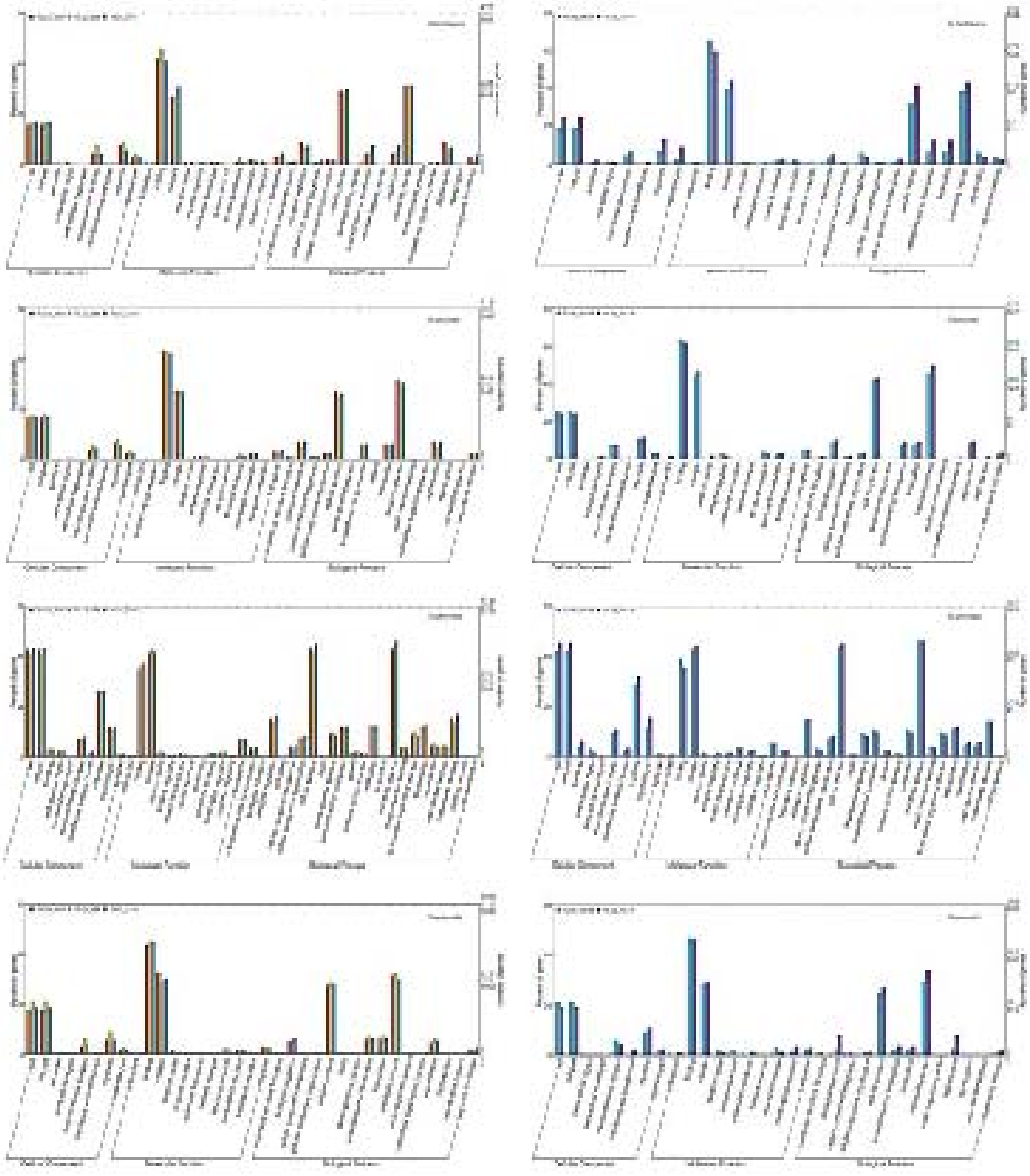

Figure S1. GO clustering analysis of the genes around (upstream 20kb and downstream 20kb) 5 selected LTR retrotransposon families.

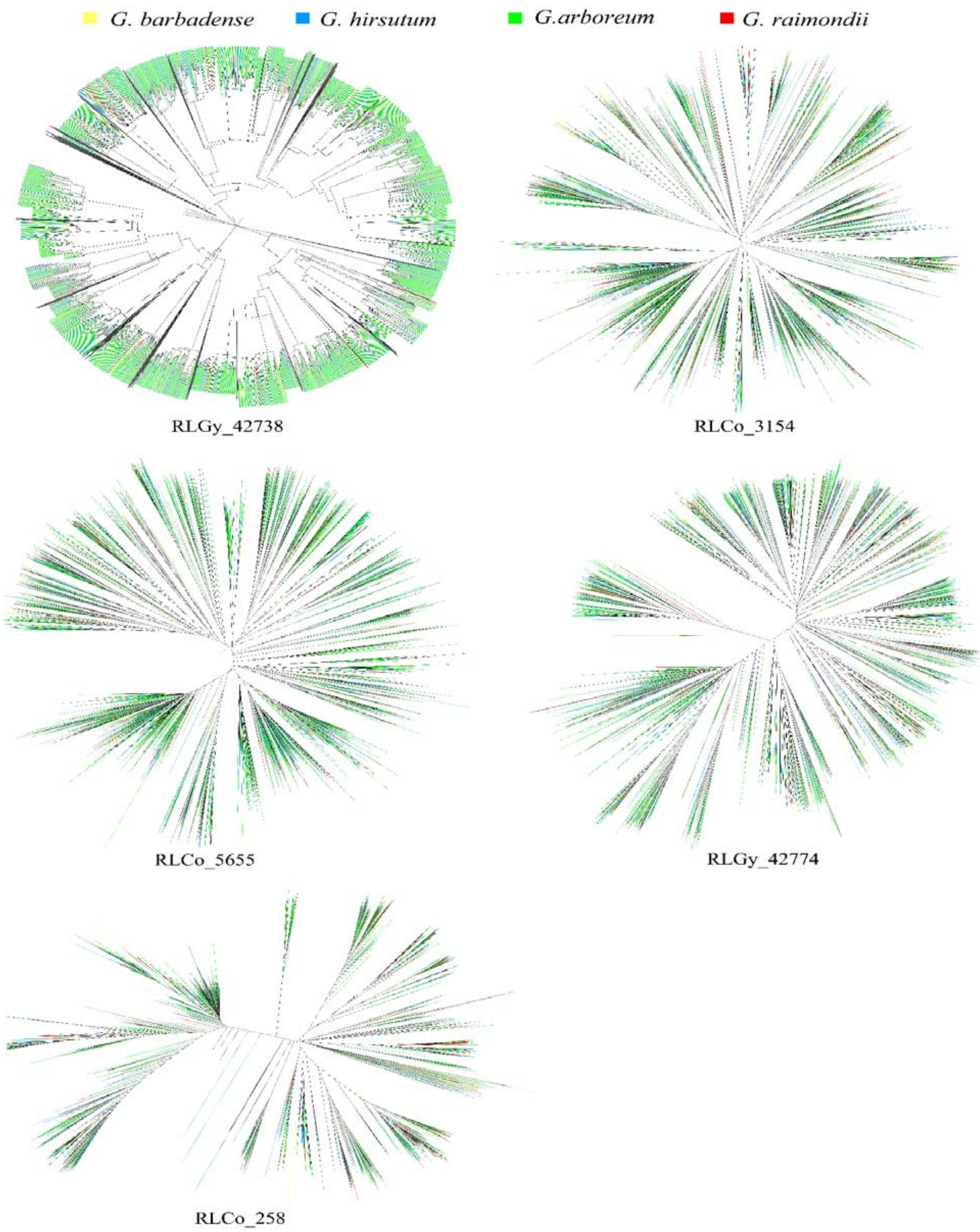

Figure S2. Phylogenetic analysis of LTR retrotransposon elements. The trees were constructed by all copies of a family from different *Gossypium* genomes analyzed.
